# Defibrillation in a newborn immediately post‐birth for severe asphyxia

**DOI:** 10.1111/ped.70163

**Published:** 2025-08-17

**Authors:** Yasuo Kitagishi, Kohei Takashima, Masahito Yamamoto, Yoshihiro Maruo

**Affiliations:** ^1^ Department of Pediatrics Shiga University of Medical Science Otsu Shiga Japan; ^2^ Department of Pediatrics Nagahama Red Cross Hospital Nagahama Shiga Japan

**Keywords:** cardiac arrest, defibrillation, electrocardiography, neonatal resuscitation, ventricular tachycardia

Since 2015, neonatal resuscitation guidelines have recommended the use of monitor electrocardiograms (ECGs) for rapid and accurate heart rate assessment.^1^ However, the interpretation of monitor ECGs and interventions, including direct current defibrillation, have not been mentioned in neonatal resuscitation guidelines. We report a case in which pulseless ventricular tachycardia (pVT) was detected by monitor ECG during resuscitation for severe neonatal asphyxia, and the infant was successfully resuscitated using defibrillation.

The patient was a female neonate born at 36 weeks and 5 days of gestation, weighing 2878 g, with severe neonatal asphyxia. The mother was a 26‐year‐old gravida 3, para 0 woman with a history of two spontaneous abortions and no significant medical history. There was no family history of sudden death or arrhythmias. No fetal cardiac anomalies or arrhythmias were noted during pregnancy. The mother presented with sudden abdominal pain and underwent an emergency cesarean section because of fetal distress. Apgar scores were recorded as 0 at 1, 5, and 10 min. Immediate resuscitation measures included chest compressions and mask ventilation, followed by endotracheal intubation at 2 min of life. Chest compressions and ventilation at a ratio of 3:1 were continued until return of spontaneous circulation (ROSC) was achieved. ECG monitoring was initiated at 9 min after birth, and the initial waveform was consistent with pulseless electrical activity (PEA). Adrenaline was administered a total of seven times (three doses of 0.07 mg/kg intratracheally and four doses of 0.02 mg/kg intravenously). At 22 min, pVT was confirmed (Figure 1). As the patient was a neonate, the defibrillation pads were placed in the anteroposterior position, with one pad on the chest and the other on the back. Defibrillation was performed with escalating energy levels: 5 J (1.8 J/kg) at 35 min, 7 J (2.5 J/kg) at 42 min, and 10 J (3.6 J/kg) at 49 min. ROSC was observed 56 min after birth. Therapeutic hypothermia was initiated after admission to the neonatal intensive care unit. However, due to worsening pulmonary hypertension 24 h later, hypothermia was discontinued, and inhaled nitric oxide (iNO) and circulatory support drugs were administered. The patient's respiratory and circulatory conditions gradually improved, leading to the discontinuation of iNO on day 11 and circulatory drugs on day 14. Brain MRI on day 17 revealed extensive cerebral edema and basal ganglia damage, indicating severe central nervous system injury. Extubation was performed on day 36, and the neonate was transitioned to high‐flow nasal cannula (HFNC) oxygen therapy. The patient was discharged on day 170, requiring HFNC respiratory support and tube feeding. Echocardiography revealed no congenital heart defects, and no arrhythmias were noted on the monitor or 12‐lead ECG from birth to discharge. Placental pathology suggested neonatal asphyxia, chorioamnionitis, capillary proliferation within the placenta, infarction at the placental margin, and inflammatory cell infiltration from the decidua to the chorion.

**FIGURE 1:**
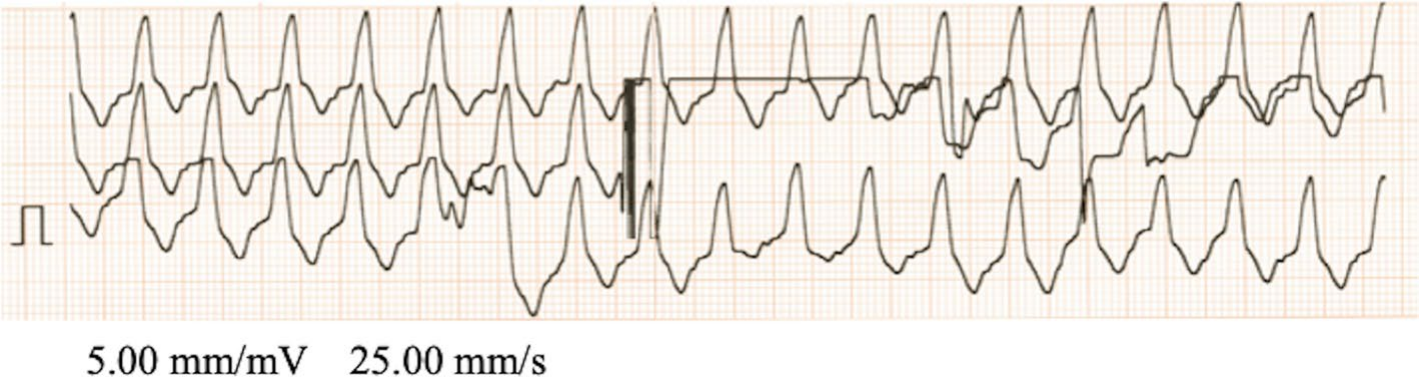
Monitor electrocardiogram waveform with pulseless ventricular tachycardia.

This case highlights successful defibrillation for pVT during severe neonatal asphyxia. Reports on defibrillation in neonatal resuscitation are extremely rare.^2^, ^3^ Previous cases have attributed shockable rhythms to maternal drug administration (local anesthetics) or underlying maternal conditions (long QT syndrome). In this case, no underlying conditions or postnatal arrhythmias were observed, suggesting that pVT developed during the resuscitation process. There are no recommendations for defibrillation energy levels in ventricular arrhythmias during neonatal resuscitation. Previous reports have achieved ROSC of less than 1.5 J/kg, suggesting that lower energy levels may be effective for neonates.^2^, ^3^ However, Pediatric Advanced Life Support (PALS) guidelines from the American Heart Association recommend an initial dose of 2 and 4 J/kg for the second shock and up to 10 J/kg for subsequent shocks for pVT and ventricular fibrillation. A systematic review of defibrillation for neonates not immediately post‐birth reported cases of defibrillation at 5 J/kg for ventricular fibrillation.^4^ Although there have been reports of successful defibrillation in neonates using low energy levels of less than 1.5 J/kg, other cases have required multiple shocks at levels below 2 J/kg before achieving ROSC. In the present case, 3.6 J/kg was required to achieve ROSC. These findings suggest that an energy level equivalent to the initial shock dose recommended in the PALS guidelines for children (2 J/kg) may also be appropriate and effective in neonatal resuscitation. Neonatal asphyxia accounts for approximately 10% of early neonatal deaths in Japan. This case of pVT during neonatal resuscitation underscores the importance of recognizing and addressing shockable rhythms. The detection of shockable rhythms is expected to increase with the guideline recommendations for monitoring ECGs in neonatal resuscitation. However, resistance to defibrillation among neonatologists has been reported in the United States.^5^ Accumulation of cases and enhanced defibrillation training for neonatologists are essential for optimizing neonatal resuscitation. In addition to educating neonatologists, it is important to ensure that defibrillators are consistently available in obstetric wards and that ECG monitoring is routinely performed to enable the timely detection of shockable rhythms. Furthermore, as resuscitation may also be initiated in obstetric wards, defibrillation training should be provided not only to neonatologists but also to all staff involved in neonatal resuscitation. In this case, defibrillation pads were placed in the anteroposterior position, which is generally considered optimal in neonates as it facilitates effective current flow through the myocardium. This configuration is suitable for their small body size, whereas the anterolateral position, commonly used in adults, may lead to pad overlap and reduced shock efficacy. Although some guidelines recommend considering the discontinuation of resuscitation after 20 min without signs of life, in Japan, resuscitation is often continued if there are any indications of possible recovery.^6^ In the present case, the appearance of pVT at 22 min of life was considered an actionable rhythm, which supported the decision to continue resuscitation beyond 20 min. The interval between defibrillations in this case was approximately 7 min. Several factors contributed to this timing, including limited staffing during the resuscitation, lack of familiarity with neonatal defibrillation due to its rarity, and the absence of specific procedural guidance in current neonatal resuscitation guidelines. These factors necessitated careful rhythm reassessment and team coordination before each shock.

## AUTHOR CONTRIBUTIONS

Conceptualization: Y.K. and M.Y.; writing—original draft: Y.K. and K.T.; formal analysis: Y.K.; writing—review and editing: Y.M. All the authors have read and agreed with the final version of the manuscript.

## CONFLICT OF INTEREST STATEMENT

The authors declare no conflict of interest.

## INFORMED CONSENT

The authors obtained written informed consent for publication of this case signed by the patient‘s parents.
